# Genome-wide DNA methylation analysis reveals that mouse chemical iPSCs have closer epigenetic features to mESCs than OSKM-integrated iPSCs

**DOI:** 10.1038/s41419-017-0234-x

**Published:** 2018-02-07

**Authors:** Wangfang Ping, Jian Hu, Gongcheng Hu, Yawei Song, Qing Xia, Mingze Yao, Shixin Gong, Cizhong Jiang, Hongjie Yao

**Affiliations:** 1 CAS Key Laboratory of Regenerative Biology, Joint School of Life Sciences, CAS Center for Excellence in Molecular Cell Science, Guangzhou Institutes of Biomedicine and Health, Chinese Academy of Sciences, Guangzhou Medical University, Guangzhou, China; 2 Institute of Health Sciences, Anhui University, Hefei, China; 3 Guangdong Provincial Key Laboratory of Stem Cell and Regenerative Medicine, Guangzhou Institutes of Biomedicine and Health, Chinese Academy of Sciences, Guangzhou, China; 4 The School of Life Sciences and Technology, Shanghai Key Laboratory of Signaling and Disease Research, The Collaborative Innovation Center for Brain Science, Tongji University, Shanghai, China

## Abstract

Induced pluripotent stem cells can be derived from somatic cells through ectopic expression of transcription factors or chemical cocktails. Chemical iPSCs (C-iPSCs) and OSKM-iPSCs (4F-iPSCs) have been suggested to have similar characteristics to mouse embryonic stem cells (mESCs). However, their epigenetic equivalence remains incompletely understood throughout the genome. In this study, we have generated mouse C-iPSCs and 4F-iPSCs, and further compared the genome-wide DNA methylomes of C-iPSCs, 4F-iPSCs, and mESCs that were maintained in 2i and LIF. Three pluripotent stem cells tend to be low methylated overall, however, DNA methylations in some specific regions (such as retrotransposons) are cell type-specific. Importantly, C-iPSCs are more hypomethylated than 4F-iPSCs. Bisulfite sequencing indicated that DNA methylation status in several known imprinted clusters, such as: *Dlk1-Dio3* and *Peg12-Ube3a*, in C-iPSCs are closer to those of mESCs than 4F-iPSCs. Overall, our data demonstrate the reprogramming methods-dependent epigenetic differences of C-iPSCs and 4F-iPSCs and reveal that C-iPSCs are more hypomethylated than OSKM-integrated iPSCs.

## Introduction

Embryonic stem cells (ESCs) and induced pluripotent stem cells (iPSCs) have great therapeutic potential for regenerative medicine and new drug screening^1^. Induced pluripotent stem cells can be achieved by several techniques, such as somatic cell nuclear transfer, cell fusion, exogenous transfection of transcriptional factors, and small-molecule treatment^2–5^. However, there are concerns that reprogramming may introduce subtle specific defects that could impact the safety of iPSCs^6,7^. Similar arguments continue as to whether these reprogrammed cells are completely equivalent in both function and character to genuine ESCs^8^. Previous studies have shown that the global expression patterns and epigenetic modifications were very similar between ESCs and 4F-induced iPSCs^9,10^, but there are yet some key differences, such as 4F-iPSCs have a unique gene expression signature, including microRNAs (miRNAs) and long non-coding RNAs^11,12^. Besides, 4F-iPSCs have genomic copy-number variations^13^. And the reactivation of c-Myc can cause tumor formation in chimeric mice derived from 4F-iPSCs^14^.

Epigenetic differences have also been observed between 4F-iPSCs and ESCs. DNA methylation is an important epigenetic modification in regulation of gene expression, genomic stability, X chromosome inactivation, and genomic imprinting. It is regarded as an obstacle in somatic cell reprogramming^15,16^, and there are strong hints that DNA methylation is altered in 4F-iPSCs. For example, differential DNA methylation distinguishes human iPSCs and hESCs^6,17^. DNA hypermethylation in 4F-iPSCs reduces their ability to differentiate toward a hematopoietic cell fate^18^. Similarly, abnormal DNA hypermethylation in 4F-iPSCs leads to aberrant silencing at the *Dlk1–Dio3* imprinted gene cluster, which decreases the developmental potential of 4F-iPSCs into chimaeric and 4n complementation-competent-iPSCs mice^19^. These epigenetic abnormalities remain a concern for the utility and safety of 4F-iPSCs in regenerative and clinical medicine.

Small chemical molecules can regulate cell signaling pathways and the epigenetic status of cells to control cell fates^20^. For example, the small molecules valproic acid (VPA, a histone deacetylase inhibitor) and vitamin C (Vc) have both been reported to prevent the abnormal silencing of the *Dlk1-Dio3* locus in 4F-iPSCs^19,21^. Chemical iPSCs (C-iPSCs)^4,22,23^ and 4F-iPSCs have been suggested to have similar characteristics to mouse embryonic stem cells (mESC). However, their epigenetic equivalence remains incompletely unclear.

Here we investigated epigenetic DNA methylation features of three different pluripotent stem cells (C-iPSCs, 4F-iPSCs, and mESCs). Our results reveal that C-iPSCs are globally hypomethylated compared to 4F-iPSCs and the DNA methylation status of imprinted regions of mESCs is closer to that of C-iPSCs than 4F-iPSCs.

## Results

### C-iPSCs, 4F-iPSCs, and mESCs all share a similar DNA methylome globally

To profile DNA methylation in C-iPSCs, we first generated C-iPSCs (Supplementary Figure 1a) from mouse embryonic fibroblasts (MEFs) as previously reported^22^. The established C-iPSC lines expressed high levels of pluripotency marker genes, such as *Oct4* and *Nanog* (Supplementary Figure 1b). The gene expression levels of pluripotency markers and DNA methylation status at *Nanog* promoters were similar among C-iPSCs, mESCs and 4F-iPSCs, which were induced by Yamanaka factors (Supplementary Figure 1c and d). To gain insight into the DNA methylomes of C-iPSCs, 4F-iPSCs, and mESCs, we performed reduced representation bisulfite sequencing (RRBS)^24^, which interrogated 1,106,981 CpG sites.

**Fig. 1:**
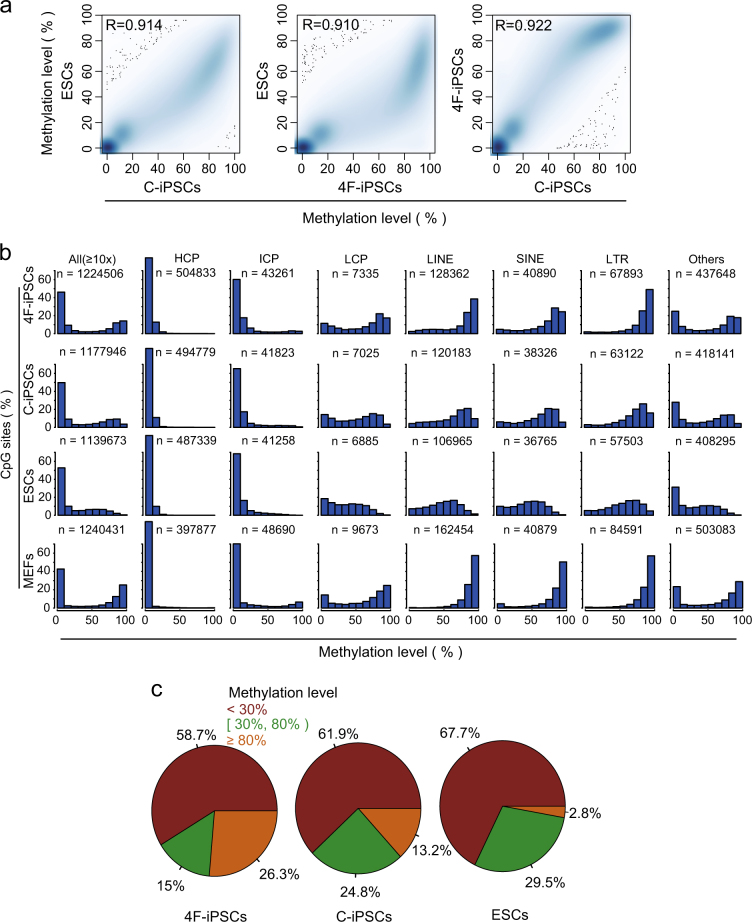
Profiles of CpG methylation in mESCs, C-iPSCs, and 4F-iPSCs for CpGs with ≥10-fold coverage. **a** Density scatter plots of CpG methylation levels between mESCs, C-iPSCs, and 4F-iPSCs. Spearman’s *R* is indicated. **b** Histograms showing the distribution of CpG methylation levels (%) in mESCs, C-iPSCs, 4F-iPSCs, and MEFs across the entire genome (All), promoters with different CpG density (HCPs, ICPs, and LCPs), interspersed repeat elements (LINEs, SINEs, and LTRs) and other genomic regions excluding promters and LINEs, SINEs, and LTRs. **c** Pie charts representing the frequency of CpGs grouped by methylation levels

The global DNA methylation profiles were highly reproducible between replicates (*R* > 0.9, Supplementary Figure 1e) and highly correlated between these cells derived using different methods (*R* > 0.922, Fig. 1a). DNA methylation levels were generally low in all three pluripotent cell types compared with MEFs, but prominently bimodal in 4F-iPSCs (Fig. 1b). The bimodality was less prominent in C-iPSCs and was absent in mESCs (Fig. 1b). To explore this bimodality in detail, we broke down the CpG sites into different classes (Fig. 1b). The results indicated that the methylation levels of high-CpG-density promoters (HCP) and intermediate-CpG-density promoters (ICP) were near-indistinguishable among the three pluripotent stem cells, even in MEFs, whereas the profiles in low-CpG-density promoters (LCP) were closer between mESCs and C-iPSCs than to 4F-iPSCs and MEFs (Fig. 1b). In addition, LCP methylations in both 4F-iPSCs and MEFs were bimodal (Fig. 1b). An important class of DNA that is methylated is the long/short-interspersed repeat elements (LINEs, SINEs) and long-terminal repeats (LTRs) that are generally suppressed by DNA methylation in somatic cells, and are mostly demethylated and active in both the reprogramming cells and mESCs^25–27^. Analysis of our DNA methylation data indicated a substantial difference in the methylation of LINEs, SINEs, and LTRs and they were all hypermethylated in 4F-iPSCs, which is similar to MEFs, but showed demetylation in mESCs and C-iPSCs (Fig. 1b).

We further examined the distribution of all CpGs grouped by methylation levels: high (≥0.8), intermediate (≥0.3 and <0.8), or low (<0.3). There was no significant difference in intermediate methylation levels, but the low and high methylations showed cell-specificity, emphasizing this change in bimodality. Notably, 4F-iPSCs were globally hypermethylated compared to mESCs (26.3% in 4F-iPSCs vs. 2.8% in mESCs) and C-iPSCs were in between (13.2%) (Fig. 1c). Taken together, iPSCs and mESCs overall share a globally similar DNA methylome with highest portion of hypermethylated CpGs in 4F-iPSCs, second in C-iPSCs, and least in mESCs.

### C-iPSCs are more DNA hypomethylated than 4F-iPSCs in a genome-wide scale

**Fig. 2:**
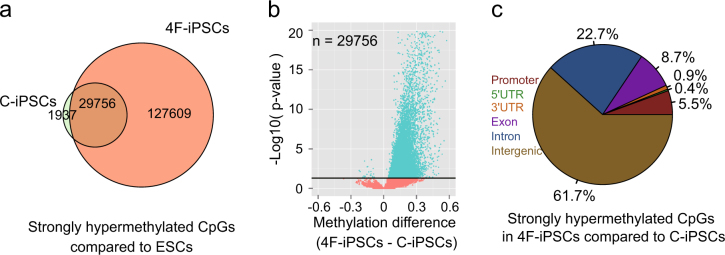
Comparison of DNA methylation levels between C-iPSCs and 4F-iPSCs. **a** Venn diagram showing the overlap between strong hypermethylated CpGs (33.3% higher than mESCs) in C-iPSCs and 4F-iPSCs. **b** Volcano plot showing the differences in methylation level (4F-iPSCs–C-iPSCs) of the common strong hypermethylated CpGs in C-iPSCs and 4F-iPSCs (the overlapped ones in **a**). *P* values are calculated by Fisher’s exact test. The black horizontal line indicates *p* value = 0.05. **c** Pie chart representing the strong hypermethylated CpGs in 4F-iPSCs compared to C-iPSCs (increased ≥33.3%) with respect to their genomic locations

We next identified 31,693 (about 3% of all CpGs) and 157,365 (about 14% of all CpGs) strongly hypermethylated CpG sites in C-iPSCs and 4F-iPSCs compared with mESCs, respectively (for strongly hypermethylated sites, the differences of methylation level were set as >0.333. Fisher’s *t* test *p* value <0.05 according to Model-based Analysis of Bisulfite Sequencing data (MOABS)). Most of the strongly hypermethylated CpG sites were common between C-iPSCs and 4F-iPSCs (Fig. 2a). This suggested that mESCs were closer to C-iPSCs than 4F-iPSCs, at least from the perspective of the strongly hypermethylated CpGs. Comparison of the common 29,756 strongly hypermethylated CpGs showed that the DNA methylation levels were significantly higher in 4F-iPSCs than in C-iPSCs (Fig. 2b). We further compared the DNA methylomes of 4F-iPSCs and C-iPSCs, and identified 14,749 CpGs and 593 differentially methylated regions (DMRs) that were strongly hypermethylated in 4F-iPSCs, and only 311 CpGs and 21 DMRs that were strongly hypomethylated in 4F-iPSCs (Supplementary Figure 2a). This confirmed 4F-iPSCs had higher levels of DNA methylation compared to C-iPSCs. The strongly hypermethylated CpGs of each cell type were largely located in intergenic and intronic regions (Fig. 2c and Supplementary Figure 2b), in agreement with the bimodality seen in the LINEs/SINEs and LTRs (Fig. 1b), which are often intergenic.

### Promoter methylation levels in C-iPSCs but not in 4F-iPSCs resemble those in mESCs

**Fig. 3:**
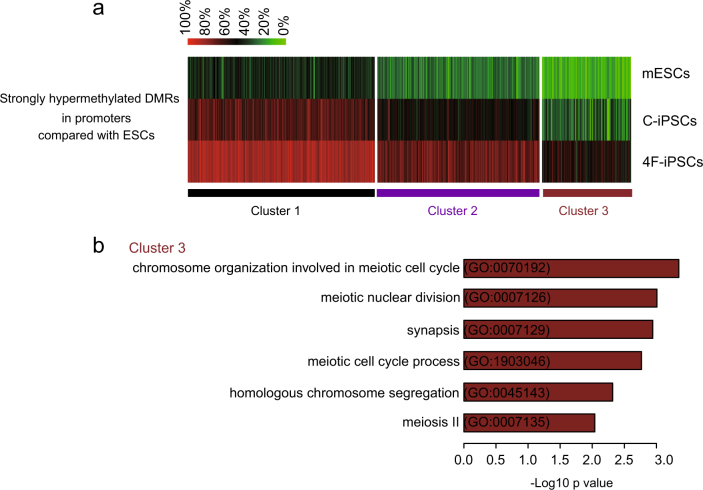
Clustering of promoter-DMRs methylation levels among C-iPSCs, 4F-iPSCs, and mESCs. **a** A methylation ratio (%) heatmap in each sample of all the strongly hypermethylated DMRs within promoter regions in C-iPSCs and 4F-iPSCs compared with mESCs. *K*-means (*K* = 3) clustering is performed. **b** Gene ontology (GO) terms associated with the genes within cluster 3 in **a**

DNA methylation of gene promoters is associated with chromatin configuration and gene expression. So we further compared DMRs of promoter regions among 4F-iPSCs, C-iPSCs, and mESCs. Compared with mESCs, strongly hypermethylated DMRs were clustered into three classes (Fig. 3a). The class 1 showed that DNA methylation levels (>80%) in C-iPSCs and 4F-iPSCs were both higher than the levels in mESCs. The class 2 showed that 4F-iPSCs (around 80%) were hypermethylated compared to mESCs and C-iPSCs (around 50%) were in between. The DNA methylation levels in class 3 of C-iPSCs were closer to mESCs compared with those in 4F-iPSCs. GO analysis indicated that the genes that have the similar DNA hypomethylation levels between C-iPSCs and mESCs were involved in chromosome organization during meiotic cell cycle, meiotic nuclear division, and synapsis (Fig. 3b).

**Fig. 4:**
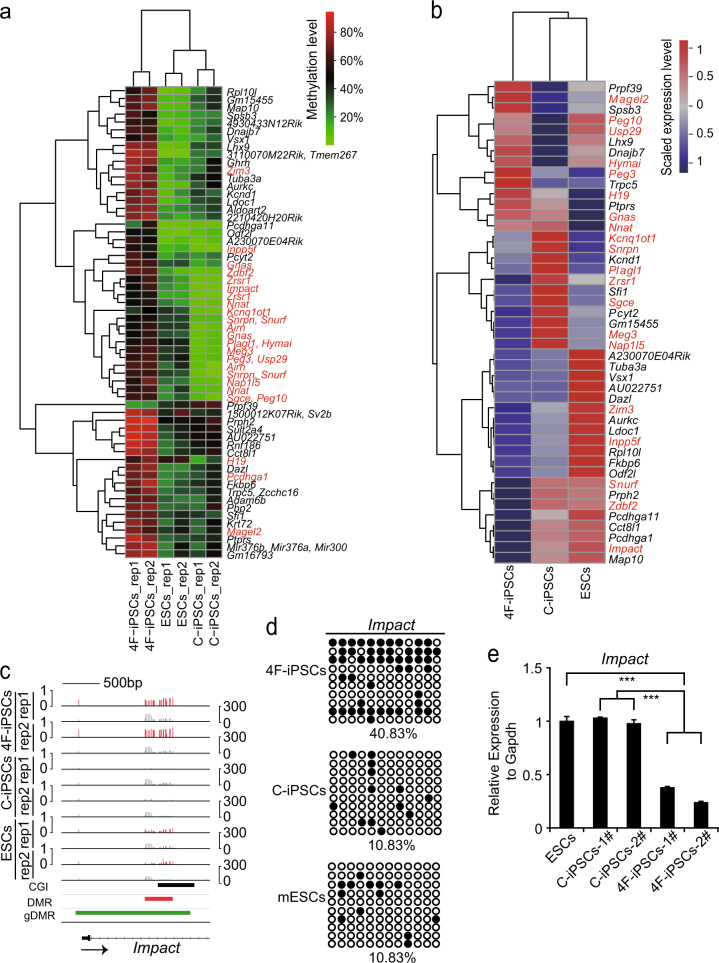
The effects of DNA methylation in promoter DMRs on gene expression among C-iPSCs, 4F-iPSCs, and mESCs. **a** Hierarchical clustering of methylation in the promoter DMRs between 4F-iPSCs and C-iPSCs. The red fonts are imprinted genes. **b** Hierarchical clustering of the expression profiles of genes associated with the DMRs in **a**. The red fonts are imprinted genes. **c** Representative DNA methylation profiles of the imprinted gene *Impact*. Red vertical lines are DNA methylation levels. Gray vertical lines are sequenced cytosine counts. CGIs from UCSC genome browser, DMRs identified in this study, and the published germline DMRs (gDMRs) are shown at the bottom. The arrow indicates the transcription direction of *Impact*. **d** Bisulfite sequencing analysis of DMR within *Impact* as labeled in **c**. Each open and filled circle represents a methylated and non-methylated CpG, respectively. The percentage of DNA methylation is shown. **e** Expression levels (qRT-PCR) of *Impact* in the three cell types. The data are presented as the mean ± SEM of experiments performed in triplicate (***p value <0.001, Student’s *t* test)

To gain insights into the difference of promoter methylation levels between the induced pluripotent cell types, we profiled DNA methylation and compared the DMRs of gene promoters between 4F-iPSCs and C-iPSCs. Clustering analysis showed that methylation levels in the DMRs of these promoters were similar between C-iPSCs and mESCs (Fig. 4a). Profiling analysis of the expression levels of the corresponding genes also clustered C-iPSCs and mESCs together (Fig. 4b). Moreover, most those genes that were repressed in 4F-iPSCs wereactivated in C-iPSCs and mESCs. Of particular interest is *Impact*, a gene preferentially expressed in neurons and modulating neurite outgrowth^28^. *Impact* was hypermethylated in 4F-iPSCs but hypomethylated in C-iPSCs and mESCs (Fig. 4c), which were confirmed by bisulfite sequencing (BS-seq) (Fig. 4d). Quantitative Real-time Polymerase Chain Reaction (qRT-PCR) analysis showed that the expression of *Impact* was significantly decreased in 4F-iPSCs (Fig. 4e). In addition, we observed similar correlations between gene expression and methylation status in promoter DMRs in the imprinted *Snrpn/Snurf* loci (Supplementary Figure 3a–c). However, a small group of genes including *Magel2*, *Trpc5*, and so on showed a methylated promoter, but were expressed, while *Prpf39* was demethylated in 4F-iPSCs but methylated in mESCs and C-iPSCs and was corrsepondingly expressed only in 4F-iPSCs (Fig. 4a, b).

### The DNA methylation and transcription profiles of imprinted genes in C-iPSCs are more similar to mESCs than 4F-iPSCs

**Fig. 5:**
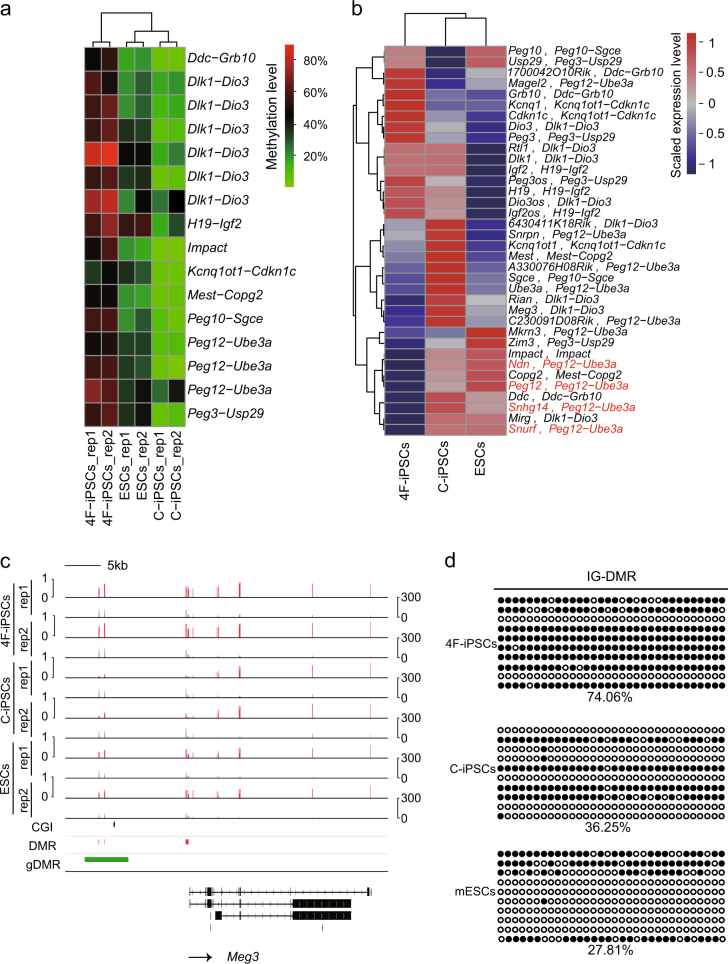
The effects of DNA methylation in DMRs within imprinting clusters on gene expression among C-iPSCs, 4F-iPSCs, and mESCs. **a** Hierarchical clustering of methylation levels in DMRs within imprinting regions between 4F-iPSCs and C-iPSCs. The names of imprinted gene clusters where DMRs locate are indicated on the right. **b** Hierarchical clustering of the expression profiles of the genes within the imprinted gene clusters. The names of the imprinted gene clusters where the genes are located are indicated after the comma. The four genes in red are used in the model schema (Fig. 6). **c** The methylation levels of imprinting control region near *Meg3* locus. Red vertical lines are DNA methylation levels. Gray vertical lines are sequenced cytosine counts. CGIs from UCSC genome browser, DMRs identified in this study, and the published germline DMRs (gDMRs) are shown at the bottom. The arrow indicates the transcription direction of *Meg3*. **d** Bisulfite sequencing analysis of IG-DMR regulating expression of *Meg3* and *Mirg* shown in Supplementary Figure 4. Each open and filled circle represents a methylated and non-methylated CpG, respectively. The percentage of DNA methylation is shown

We noticed that, among our list of DMR genes, there were several well-known imprinted genes, such as *H19*,* Peg10*, and *Impact*. The murine genome contains around 150 imprinted genes that are typically located in clusters^29^. Importantly, imprinted genes have important roles in mammalian development^30^. Therefore, we next focused on the DNA methylation and transcription levels of these imprinted genes. We calculated the methylation levels in the known imprinted clusters that spread over 20 kb–3.7 Mb of DNA^31^, such as the *Dlk1-Dio3*, *Peg12-Ube3a*, *H19-Igf2*, *Mest-Copg2*, *Ddc-Grb10*, *Peg3-Usp9*, *Kcnq1ot1−Cdkn1c* loci. The results indicated that mESCs and C-iPSCs showed similar hypomethylation levels in the DMRs, whereas 4F-iPSCs were hypermethylated (Fig. 5a). Consistently, expression profiles of the genes within these imprinting regions clustered mESCs and C-iPSCs together (Fig. 5b). The *Dlk1-Dio3* imprinting cluster was hypermethylated in 4F-iPSCs. In contrast, this cluster was hypomethylated in C-iPSCs and mESCs (Fig. 5a, c). The cluster contains the intergenic germline-derived DMR (IG-DMR), whose methylation patterns are established in the germline. The IG-DMR, located 70 kb downstream of *Dlk1* to 15 kb upstream of *Gtl2*, is believed to be a control element for this imprinted gene cluster^32,33^. The methylation levels of IG-DMR in C-iPSCs resembled those of mESCs, but those in 4F-iPSCs were the highest, which was confirmed by BS-seq (Fig. 5d). Correspondingly, the genes in *Dlk1-Dio3* cluster were aberrantly silent in 4F-iPSCs, including *Meg3* and *Mirg* (Supplementary Figure 4a and b). What’s more, validated germline DMRs (gDMRs), which usually function as imprinting control elements, were hypermethylated in 4F-iPSCs and hypomethylated in mESCs and C-iPSCs (Supplementary Figure 5).

Aberrant methylation in DMRs in the imprinted clusters can lead to diseases such as Prader–Willi syndrome (PWS), a complex neurogenetic disorder, caused by loss of expression of paternally imprinted genes located on human chromosome 15q11-q13^34,35^. The PWS imprinted cluster in human has its orthologous locus on mouse chromosome 7C, that is, the *Peg12-Ube3a* cluster^36^. In addition, multiple studies have identified the 5′ untranslated region of the *Snrpn* gene, which has been identified as the PWS Imprinting Center (IC).

**Fig. 6:**
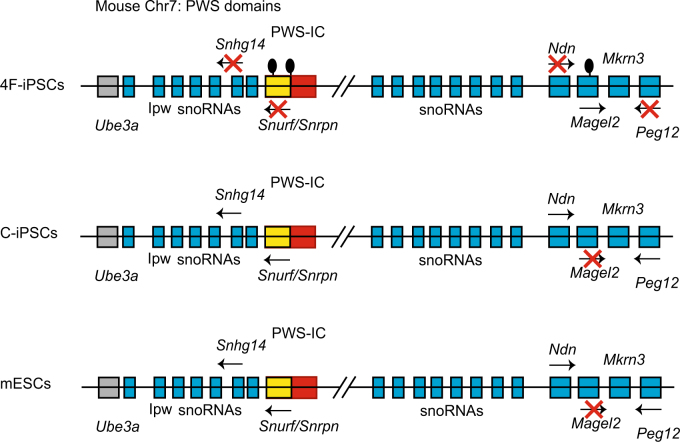
Schematic diagrams of mouse *Peg12-Ube3a* imprinting cluster among 4F-iPSCs, C-iPSCs, and mESCs.

Our data showed that all three DMRs within *Peg12-Ube3a* cluster were hypermethylated in 4F-iPSCs (Fig. 5a) and consistently, their target genes *Snhg14*, *Snurf/Snrpn*, *Ndn*, and *Peg12* were silenced in 4F-iPSCs (Fig. 5b). In contrast, interestingly, the DMRs in *Snrpn/Snurf* loci were hypomethylated in C-iPSCs and mESCs (Supplementary Figure 3b) and therefore, the target genes were transcribed. Contrarily, expression level of another target gene *Magel2* was positively correlated to methylation levels in the DMRs (Fig. 4a, b), implying a more complex regulatory system than just DNA methylation (Fig. 6).

## Discussion

Collectively, our studies indicated that C-iPSCs were more hypomethylated than 4F-iPSCs and their DNA methylation status was closer to mESCs than to 4F-iPSCs. Notably, this may correlate with the different development potentials of C-iPSCs and 4F-iPSCs, which are highlighted by the fact that 4F-iPSCs contribute to chimeras to a lesser extent 10–50%^19^, while C-iPSCs generate chimeras as about 100%^22^. This reduced development potential of 4F-iPSCs may be also partly explained by abnormal silencing of the specific imprinted genes.

We also noted that repeat elements (LINEs, SINEs, and LTRs) in both C-iPSCs and 4F-iPSCs tend to be hypermethylated, and especially in 4F-iPSCs, in contrast to mESCs. This hypermethylation of DNA levels might be involved in the silencing of retrotransposons in C-iPSCs and 4F-iPSCs. The role of repeat elements in pluripotency is complex and they can be both beneficial and deleterious^25^, for example many long-non-coding RNAs are derived from repeat elements and are required to maintain pluripotency^12,37^. Reprogramming mimics early embryonic development, where retroelements of specific families show embryonic stage-specific demethylation before inner cell mass stage and then de novo DNA methylation occurs, while other elements remain higher methylation levels throughout development^24^. Unlike C-iPSCs and mESCs, the methylation levels of repeat elements in 4F-iPSCs are somewhat similar to mouse somatic cells. The lack of correct DNA demethylation of repeat elements in 4F-iPSCs might imply an incomplete reprogramming.

Besides, our results showed that C-iPSCs and 4F-iPSCs have differences in the pattern of DNA methylation in non-coding regions (Fig. 1c). These non-coding regions may include promoters and distal regulatory elements, such as enhancers and transcription factor binding sites. Growing evidence suggests that cell- and tissue-specific changes in DNA methylation are associated with non-coding sequences^38–41^. Many studies have suggested that super-enhancers, which regions of the genome occupied by multiple transcription factors, cofactors, chromatin regulators, and transcription apparatus, are associated with the expression of key cell identity genes^42,43^ and many tissue-specific enhancers are hypomethylated in tissues where the target genes are expressed, but are hypermethylated in tissues where the target genes are silent^40,41^. Thus, we hypothesize that the differences in DNA methylation on non-coding regions may regulate the expression of target genes.

In addition, previous studies reported that human iPSCs created from PWS patients using the four pluripotency factors OCT4, SOX2, KLF4, and MYC retained the molecular signature of PWS including hypermethylation of SNRPN and NDN^44^. Mouse models lacking the *Snrpn/Snurf* gene showed a postnatal lethality and surviving mice showed growth retardation similar to PWS phenotype^34.^ And the *NDN* gene encodes the protein necdin and Necdin-null mouse model showed respiratory defect that was also similar to those found in PWS patients^34^. Our findings suggest that 4F-iPSCs, although mostly similar to mESCs and C-iPSCs, have abnormal DNA methylation and expression at some key imprinted genes. Thus these differences may be not only derived from the original cells, but also a widespread phenomenon. The mechanisms that might cause this imprinting disorder require further studies.

Overall, this study is the first report, which demonstrates the DNA methylation profiles of C-iPSCs in a genome-wide scale, and compares the differential DNA methylation at imprinted regions among C-iPSCs, 4F-iPSCs, and mESCs. Taken together, we provide a strong evidence that chemical reprogramming might be better than transcription factor-integrated reprogramming.

## Materials and Methods

### Mice

C57BL/6J, CBA/CaJ, and 129S4/SvJaeJ mice were purchased from the Jackson Laboratory. C57BL/6J, CBA/CaJ, and 129S4/SvJaeJ were used for generating OG2-MEFs. Animal experiments were performed according to the guidelines for the Care and Use of Laboratory Animals of the National Institutes of Health.

### Cell culture

Plat-E cells were maintained in DMEM high-glucose media (Hyclone) supplemented with 10% FBS (Excell). OG2-MEFs were maintained in Dulbecco’s high-glucose modified eagles medium (DMEM) containing 15% fetal bovine serum (FBS, Gibco), 1 mM non-essential amino acids (Gibco), 1% GlutaMAX (Gibco). Mouse E14 ESCs, 4F-iPSCs, and C-iPSCs were maintained on gelatin-coated plates in a mESC culture medium containing DMEM high glucose (Gibco), 15% FBS (Gibco), 1% GlutaMAX (Gibco), 1% non-essential amino acids (NEAA, Gibco), 1 mM sodium pyruvate (Gibco), 1% penicillin–streptomycin (Gibco), 0.055 mM 2-mercaptoethanol (Life Technologies), 1000 U/ml leukemia inhibitory factor (LIF, Millipore), and supplemented with 2i (3 μM CHIR99021 (Selleck) and 1 μM PD0325901 (Selleck)).

### C-iPSCs generation

Chemical iPSCs were generated from mouse OG2-MEFs using small-molecule cocktails, as previously described^22^. OG2-MEFs were plated 50,000 cells per well of a six-well plate. The next day (day 0), the culture was changed into stage 1 medium: knockOut DMEM (Gibco) supplemented with 10% KnockOut™ serum replacement (KSR, Gibco), 10% FBS (Gibco), 1% GlutaMAX (Gibco), 1% NEAA (Gibco), 0.055 mM 2-mercaptoethanol (Life Technologies), 1% penicillin–streptomycin (Gibco), 100 ng/ml bFGF (Origene) and the small-molecule cocktail VC6TFAE (0.5 mM VPA (Sigma), 20 μM CHIR99021 (Selleck), 10 μM 616452 (Selleck), 5 μM tranylcypromine hydrochloride (Tocris), 50 μM Forskolin (Enzo Life Sciences), 0.05 μM AM580 (Tocris), and 5 μM EPZ004777 (Selleck)). On day 12, the cells were replated at 150,000 cells per well of a six-well plate. During days 12–16, concentrations of bFGF, CHIR, and forskolin were reduced to 25 ng/ml, 10 μM, and 10 μM, respectively. On day 16, the culture was changed into stage 2 medium containing 25 ng/ml bFGF, 0.5 mM VPA, 10 mM CHIR99021, 10 mM 616452, 5 mM tranylcypromine, 10 mM forskolin, 0.05 mM AM580, 0.05 μM DZNep (Tocris), 0.5 μM 5-Aza-2′-deoxycytidine (Enzo Life Sciences), and 5 μM SGC0946 (Selleck). On day 28, the culture was transferred into stage 3 medium: mix DMEM/F12 (Invitrogen) and Neurobasal (Invitrogen) with a proportion of 1:1, 1% N2 supplement (Invitrogen), 2% B27 supplement (Invitrogen), 1% GlutaMAX (Gibco), 1% NEAA (Gibco), 0.055 mM 2-mercaptoethanol (Life Technologies), 1% penicillin–streptomycin (Gibco), 3 μM CHIR99021 (Selleck), 1 μM PD0325901 (Selleck), and 1000 U/ml leukemia inhibitory factor (LIF, Millipore). After another 8–12 days, mESC-like and green fluorescent protein-positive C-iPSC colonies emerged and were then picked up for expansion and characterization. Any medium was changed every 4 days.

### 4F-iPSCs generation

30,000 OG2-MEFs were plated in a six-well plate and then infected twice with retroviral supernatants generated with Plat-E cells. Plat-E cells were transfected pMAXs-Oct4/Sox2/Klf4/c-Myc using modified polyethylenimine. OG2-MEFs were infected with equal volumes of the four supernatants of each OSKM transcription factor containing polybrene at a final concentration of 8 μg/ml. Infected cells were cultured with mESC medium and renewed daily. Green fluorescent protein-positive 4F-iPSCs colonies appeared about day 10 post infection and were picked up day 14 for expansion.

### Immunofluorescence

Cells were washed twice with PBS and then fixed in 4% paraformaldehyde at room temperature for 20 min. After fixation, cells were treated with 0.3% Triton

X-100 in PBS containing 10% goat serum at room temperature for 15 min. Cells were then incubated with primary antibodies at 4°C overnight. The primary antibody used for cell immunofluorescence was anti-NANOG (Novus Biologicals). And then the cells were washed for three times and were incubated with corresponding secondary antibody in a cassette at room temperature for 1 h. Then the cells were washed for three times with PBS and then nuclei were stained with DAPI (Sigma). Images were captured with an inverted microscope (DMI4000, Leica Microsystems).

### Quantitative real-time PCR

Total RNA was collected using the TRIzol reagent (MRC). Complemetary DNAs were synthesized from 1 μg RNA by using First-Strand cDNA synthesis Kit (TOYOBO) with Oligo18 (dT) and Random Primer. PCR was carried out using SYBR green (Genstar) and performed on a CFX Real-Time System (Bio-Rad).

### Bisulfite sequencing

DNA was extracted using TIANamp Genomic DNA Kit (TIANGEN). Bisulfite modification of the isolated DNA was performed using EpiTect Bisulfite Kit (QIAGEN). The bisulfite-modified DNA was amplified by PCR or nested PCR using TaKaRa EpiTaq™ HS (for bisulfite-treated DNA) (Takara). To purify the PCR products, the DNA fragments were separated by electrophoresis using a 2% agarose gel. Then the bands were excised and purified with the TIANgel purification kit (TIANGEN). The PCR products were cloned into the pMD18-T Vector (Takara). Randomly picked 10 clones from each sample were sequenced.

### RNA-seq and data analysis

RNA sequencing libraries were sequenced on an Illumina Hiseq 4000 platform and 150 bp paired-end reads were generated. Sequencing reads were aligned to annotated mouse transcripts (mm10) using TopHat v2.0.13^45^. High-quality mapped read pairs were retained for evaluation of gene expression using Cuffdiff v2.2.1^46^ with default parameters. Expression levels (FPKM, fragments per kilobase per million mapped reads) for each gene were converted to a *Z*-score for hierarchical l clustering analysis. Genes with no expression in all three cell types were removed before plotting heatmaps.

### Reduced representative bisulfite sequencing and data analysis

Chemical iPSCs, 4F-iPSCs, and mouse ESCs were collected. Genomic DNA was digested with MspI (NEB) and libraries were size-selected (170–370 bp) on an agarose gel followed by bisulfite conversion. Libraries were sequenced on an Illumina HiSeq 2500 sequencer as paired-end 50-bp reads.

Sequencing reads of RRBS were aligned to mouse reference genome (mm10) using BSMAP v2.90^47^ with up to 8% mismatches. Only uniquely mapped pairs were retained for analysis of DNA methylation. MOABS v1.3.2^48^ was used to merge replicates and measure CpG methylation levels. Only the CpGs with ≥10-fold coverage in all samples were kept for differentially methylated CpG sites (DMCs) and DMR calling by mcall tool in MOABS with default parameters. Totally 1,106,981 CpGs were obtained. Strong hyper– or hypo–methylated (methylation differences >33.3%, *p* value <0.05) DMCs and DMRs were retrieved from MOABS output for further analysis according to Fisher’s exact test method.

Promoters were defined as −1 to 1 kb of the transcription start sites. Promoter classes based on CpG density (HCP, ICP, and LCP) were obtained by Bioconductor package compEpiTools v1.6.4^49^ using R v3.3.0. HCP, ICP, and LCP were defined as previously published^50^. Genomic locations of CpG islands and interspersed repeat families (LINEs, SINEs, LTRs) were downloaded from UCSC genome browser. The gDMRs were downloaded from: https://atlas.genetics.kcl.ac.uk/^51^ and converted to the coordinates of mm10 genome assembly. The DMRs that are overlapped with a promoter are defined as promoter DMRs. Imprinting-region DMRs were defined in a similar manner.

Strong hypermethylated promoter DMRs in C-iPSCs that are at least 33.3% higher than mESCs were defined strong hypermethylated promoters DMRs in C-iPSCs compared to mESCs. The strong hypermethylated promoters DMRs in 4F-iPSCs compared to mESCs were defined in the same way. We merged the overlapped DMRs from these two sets of strong hypermethylated promoters DMRs and re-calculated the methylation level of the newly merged DMRs for each cell type. The methylation level of a DMR was calculated as the averaged methylation level of CpGs within the DMR.

In order to compare all DMRs between every two samples together, overlapped DMRs were merged and methylation ratio of merged DMRs were defined as the averaged methylation ratio of CpGs within the merged DMRs. We applied *K*-means (*K* = 3) method to clustering promoter DMRs into three classes. The genes in each cluster were re-ordered by official symbol.

### Statistical analysis

Data are presented as mean values ± SD unless otherwise indicated in figure legends. For statistical comparison of two groups, we performed two-tailed Student’s *t* test. Differences in means were considered statistically significant at *p* < 0.05. Significance levels are: *p < 0.05; **p < 0.01; ***p < 0.001.

Comparisons between two CpGs or methylation regions were performed using Fisher’s exact test (*p* < 0.05 was considered significant).

### Accession numbers

The RNA-seq and RRBS data sets have been deposited in the Gene Expression Omnibus (GEO) under accession number GSE92985. MEF RRBS data sets were downloaded from GEO under accession number GSE52741^15^.

### Primers

Primers for quantitative PCR

**Table:** 

**Primers**	**Sequence** **(5**′–**3**′**)**
Oct4-forward	GGCTTCAGACTTCGCCTCC
Oct4-reverse	AACCTGAGGTCCACAGTATGC
Sox2-forward	AGGGCTGGGAGAAAGAAGAG
Sox2-reverse	CCGCGATTGTTGTGATTAGT
Nanog-forward	AAGCAGAAGATGCGGACTGT
Nanog-reverse	ATCTGCTGGAGGCTGAGGTA
Impact-forward	GTGAAGAAATCGAAGCAATGGC
Impact-reverse	GGTACTCACTTGGCAACATCA
Snurf-forward	TCCAGGTCAAACGTCGAAGG
Snurf-reverse	CGTGGGTACAAGTGACACTCTT
Meg3-forward	TTGCACATTTCCTGTGGGAC
Meg3-reverse	AAGCACCATGAGCCACTAGG
Mirg-forward	GCGGTCAACACTGGGTACTT
Mirg-reverse	CCTGAGGACCAATTCAGCGT

Primers for bisulphite sequencing analysis

**Table:** 

**Primers**	**Sequence** **(5**′–**3**′**)**
Nanog-forward	GATTTTGTAGGTGGGATTAATTGTGAATTT
Nanog-reverse	ACCAAAAAAACCCACACTCATATCAATATA
Snrpn/Snurf-outside forward	TATGTAATATGATATAGTTTAGAAATTAG
Snrpn/Snurf-outside reverse	AATAAACCCAAATCTAAAATATTTTAATC
Snrpn/Snurf-inside forward	AATTTGTGTGATGTTTGTAATTATTTGG
Snrpn/Snurf-inside reverse	ATAAAATACACTTTCACTACTAAAATCC
IG-DMR-outside forward	GTGTTAAGGTATATTATGTTAGTGTTAGG
IG-DMR-inside forward	ATATTATGTTAGTGTTAGGAAGGATTGTG
IG-DMR-reverse	TACAACCCTTCCCTCACTCCAAAAATT
Impact-forward	TGGATGAGGTGTATAATTTT
Impact-reverse	CAAAACAAAACTAAACCTAC

## Electronic supplementary material

Supplemental Legends

Supplementary Figures
