# Supplementary material for: Genome-wide DNA methylation analysis reveals that mouse chemical iPSCs have closer epigenetic features to mESCs than OSKM-integrated iPSCs
Source: Cell Death Dis. 2018 Feb 7;9(2):187. doi: 10.1038/s41419-017-0234-x (PMC5833453; doi:10.1038/s41419-017-0234-x)
Supplement: Supplementary file 1 — Supplemental Legends [file 41419_2017_234_MOESM1_ESM.docx]

**Supplementary Figure Legends**

**Supplementary Figure 1. Characterization of C-iPSCs.** **(a)** Microscopy images showing cell morphology changes during chemical reprogramming. D12, D24, and D38 indicate the 12^th^, 24^th^, and 38^th^ day of reprogramming, respectively. Scale bar represents 50 μm. **(b)** Immunofluorescence staining showing expression of NANOG and OCT4 in a representative C-iPSC cell line. Scale bar represents 50 μm. **(c)** Expression levels of representative genes in MEFs, C-iPSCs, 4F-iPSCs and mESCs. **(d)** Bisulfite genomic sequencing of Nanog promoter regions in different cell types. D16 indicates the intermediate C-iPSCs at day 16^th^ of reprogramming. Each open and filled circle represents a methylated and non-methylated CpG, respectively. The percentage of DNA methylation is shown. **(e)** Scatter plots showing high reproducibility (Spearman's R) of global methylation levels for CpGs with ≥ 10-fold coverage between two replicates.

**Supplementary Figure 2. Comparison of DNA methylation levels between C-iPSCs and 4F-iPSCs.** **(a)** Bar plots showing the frequency of differentially methylated cytosines (DMCs) and CpG regions (DMRs) between C-iPSCs and 4F-iPSCs. **(b)** Pie charts representing the strong hypermethylated (increased ≥ 33.3%) CpGs in C-iPSCs compared to mESCs (left) and in 4F-iPSCs compared to mESCs (right) with respect to their genomic locations. All CpGs are covered by ≥ 10 reads in each sample.

**Supplementary Figure 3. The effect of DNA methylation at Snurf/Snrpn loci on *Snurf* gene expression among 4F-iPSCs, C-iPSCs and mESCs.** **(a)** Representative DNA methylation profiles of imprinted gene *Snurf* and *Snrpn*. Red vertical lines are DNA methylation levels. Grey vertical lines are sequenced cytosine counts. CGIs from UCSC genome browser, DMRs identified in this study and the published germline DMRs (gDMRs) are shown at the bottom. The arrow indicates the transcription direction. **(b)** Bisulfite sequencing analysis of DMR within *Snurf* and *Snrpn* as labeled in A. Each open and filled circle represents a methylated and non-methylated CpG, respectively. The percentage of DNA methylation is shown. **(c)** Expression levels (qRT-PCR) of *Snurf* in the three cell types (***: p-value < 0.001, Student’s t-test).

**Supplementary Figure 4. Analysis of RNA expression levels of *Meg3* and *Mirg* among 4F-iPSCs, C-iPSCs and mESCs.** **(a)** Expression levels (qRT-PCR) of *Meg3* in the three cell types (*: p-value < 0.05, ***: p-value < 0.001, Student’s t-test). **(b)** Expression levels (qRT-PCR) of *Mirg* in the three cell types (**: p-value < 0.01, ***: p-value < 0.001, Student’s t-test).

**Supplementary Figure 5. Comparison of methylation levels of gDMRs among 4F-iPSCs, C-iPSCs and mESCs.** Hierarchical clustering of DNA methylation levels in the previously validated germline DMRs (gDMRs). The names of germline DMRs are indicated at right.
