# Supplementary material for: Genome-wide DNA methylation analysis reveals that mouse chemical iPSCs have closer epigenetic features to mESCs than OSKM-integrated iPSCs
Source: Cell Death Dis. 2018 Feb 7;9(2):187. doi: 10.1038/s41419-017-0234-x (PMC5833453; doi:10.1038/s41419-017-0234-x)

Supplementary Figure 1

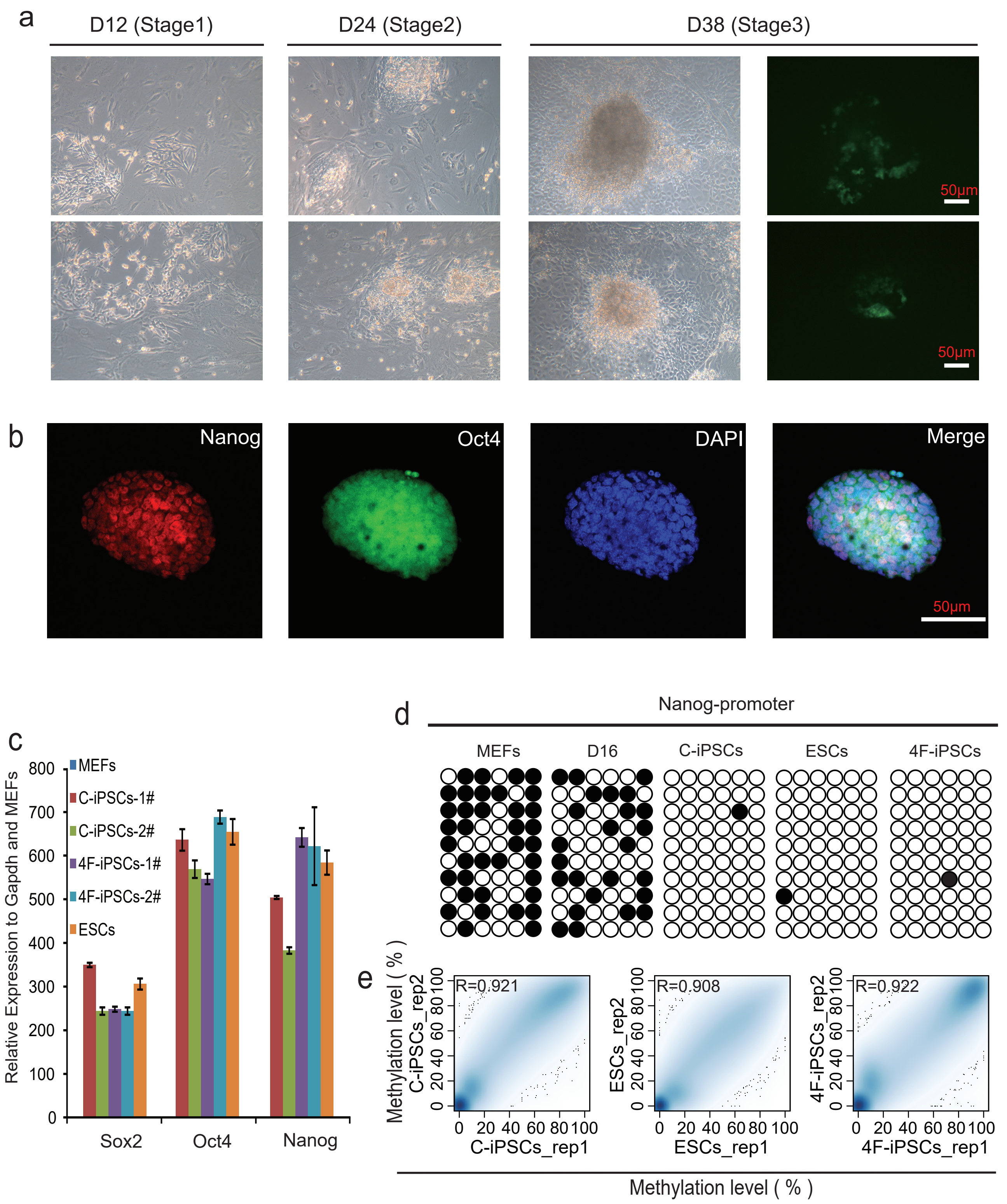

Supplementary Figure 2

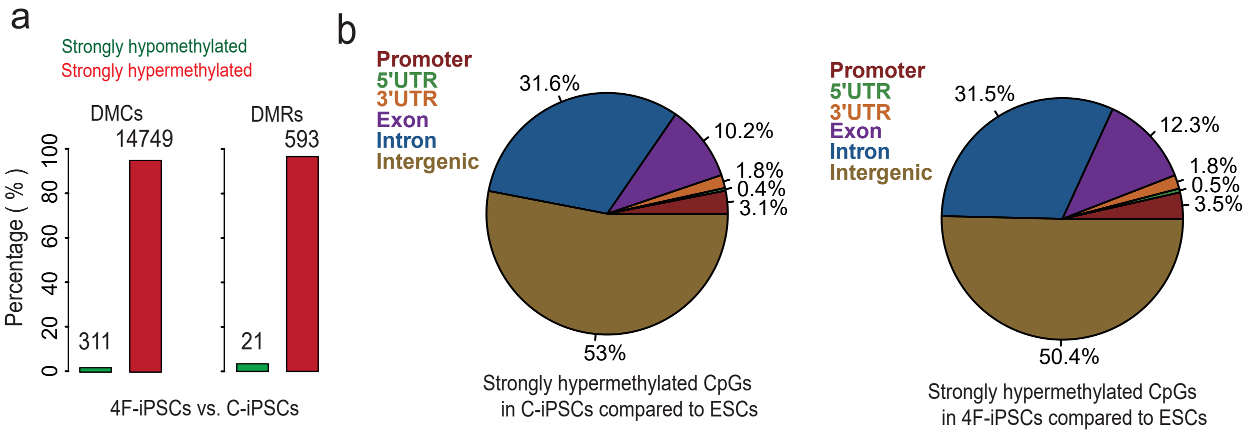

Supplementary Figure 3

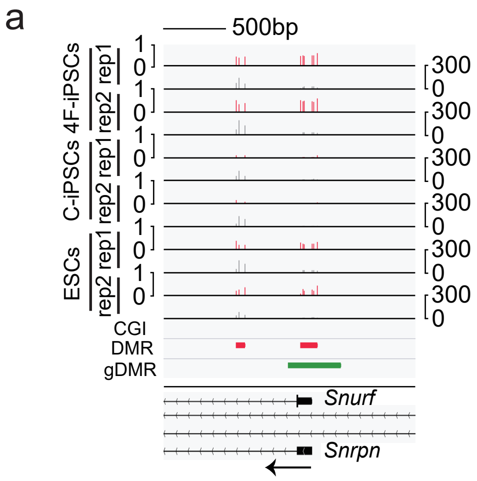

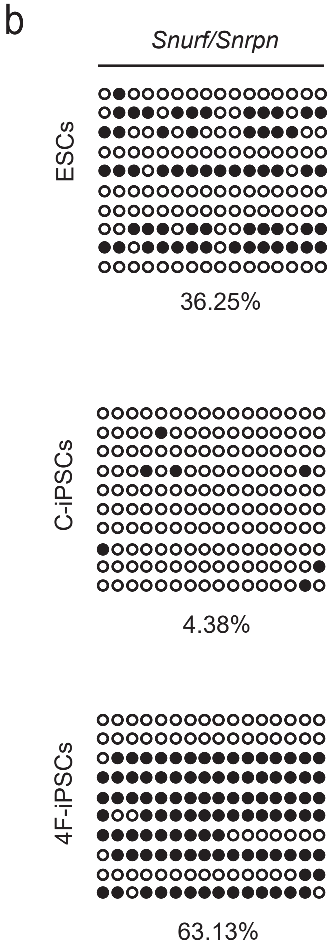

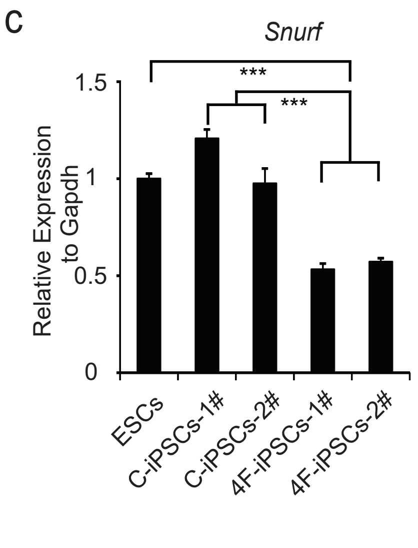

Supplementary Figure 4

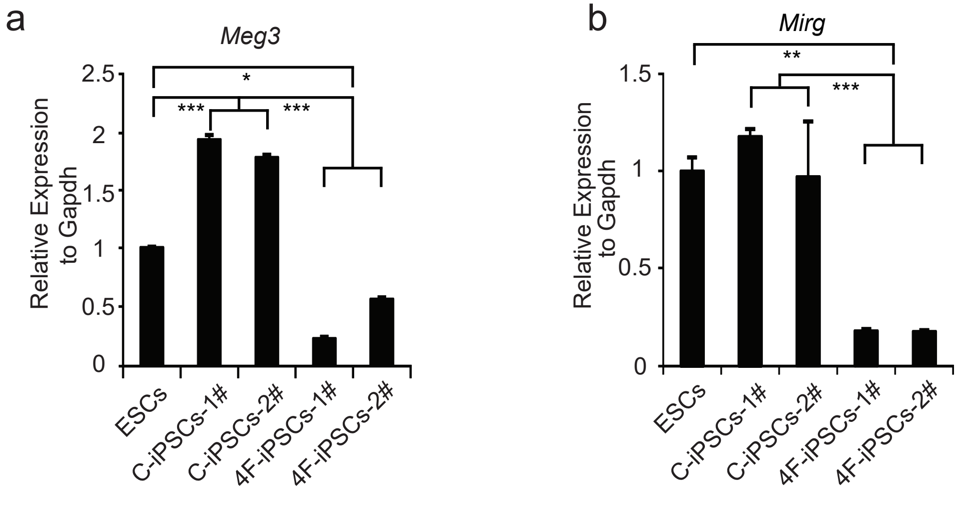

Supplementary Figure 5

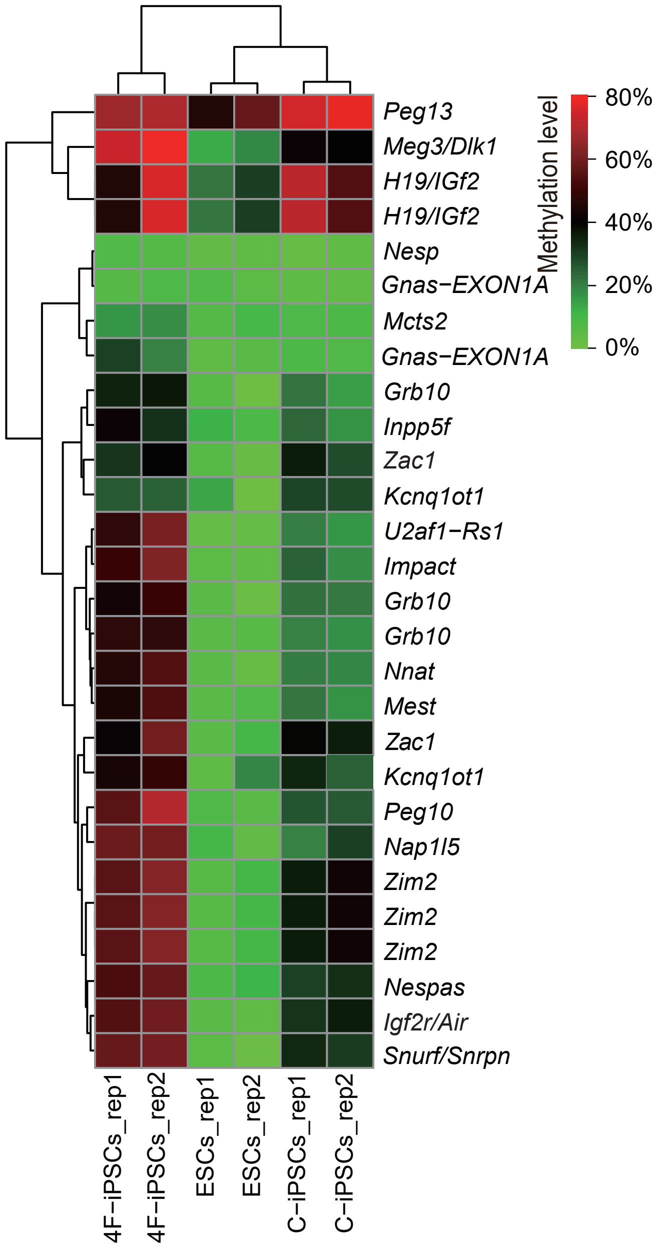

Supplement: Supplementary file 2 — Supplementary Figures [file 41419_2017_234_MOESM2_ESM.pdf]
